# Recommendations for Epstein-Barr virus–based screening for nasopharyngeal cancer in high- and intermediate-risk regions

**DOI:** 10.1093/jnci/djad012

**Published:** 2023-02-01

**Authors:** W K Jacky Lam, Ann D King, Jacob A Miller, Zhiwei Liu, Kelly J Yu, Melvin L K Chua, Brigette B Y Ma, Ming Yuan Chen, Benjamin A Pinsky, Pei-Jen Lou, John K S Woo, Wan-Lun Hsu, Julia Simon, Denise L Doolan, Tim Waterboer, Edwin P Hui, Hui Li, Raymond K Tsang, Kenneth C W Wong, Julian P Goh, Alexander C Vlantis, Qi Yong Ai, Lun M Wong, Victor Abdullah, Jin Ching Lin, Chien-Jen Chen, Ruth M Pfeiffer, Quynh-Thu Le, Anne W M Lee, Mingfang Ji, Sumei Cao, Jun Ma, Anthony T C Chan, K C Allen Chan, Allan Hildesheim

**Affiliations:** Li Ka Shing Institute of Health Sciences, The Chinese University of Hong Kong, Hong Kong SAR, China; Department of Chemical Pathology, The Chinese University of Hong Kong, Prince of Wales Hospital, Hong Kong SAR, China; Centre for Novostics, Hong Kong Science Park, Hong Kong SAR, China; State Key Laboratory of Translational Oncology, Sir YK Pao Centre for Cancer, Hong Kong Cancer Institute, The Chinese University of Hong Kong, Prince of Wales Hospital, Hong Kong SAR, China; Department of Otorhinolaryngology, Head and Neck Surgery, The Chinese University of Hong Kong, Prince of Wales Hospital, Hong Kong SAR, China; Department of Imaging and Interventional Radiology, The Chinese University of Hong Kong, Prince of Wales Hospital, Hong Kong SAR, China; Department of Radiation Oncology, Stanford University, Stanford, CA, USA; Division of Cancer Epidemiology and Genetics, National Cancer Institute, Rockville, MD, USA; Division of Cancer Epidemiology and Genetics, National Cancer Institute, Rockville, MD, USA; Department of Head and Neck and Thoracic Cancers, Division of Radiation Oncology, National Cancer Centre Singapore, Singapore, Singapore; Division of Medical Sciences, National Cancer Centre Singapore, Singapore, Singapore; Oncology Academic Programme, Duke-NUS Medical School, Singapore, Singapore; State Key Laboratory of Translational Oncology, Sir YK Pao Centre for Cancer, Hong Kong Cancer Institute, The Chinese University of Hong Kong, Prince of Wales Hospital, Hong Kong SAR, China; Department of Clinical Oncology, State Key Laboratory of Translational Oncology, Sir YK Pao Centre for Cancer, Hong Kong Cancer Institute, The Chinese University of Hong Kong, Prince of Wales Hospital, Hong Kong SAR, China; Department of Nasopharyngeal Carcinoma, Sun Yat-sen University Cancer Center, Guangzhou, Guangdong, China; State Key Laboratory of Oncology in South China, Collaborative Innovation Center for Cancer Medicine, Guangdong Key Laboratory of Nasopharyngeal Carcinoma Diagnosis and Therapy, Center for Precision Medicine of Sun Yat-sen University, Sun Yat-sen University Cancer Center, Guangzhou, Guangdong, China; Department of Pathology, Stanford University, Stanford, CA, USA; Department of Otolaryngology, National Taiwan University Hospital and College of Medicine, Taipei, Taiwan; Department of Otorhinolaryngology, Head and Neck Surgery, The Chinese University of Hong Kong, Prince of Wales Hospital, Hong Kong SAR, China; Master Program of Big Data in Biomedicine, College of Medicine, Fu Jen Catholic University, New Taipei City, Taiwan; Data Science Center, College of Medicine, Fu Jen Catholic University, New Taipei City, Taiwan; Infections and Cancer Epidemiology, Infection, Inflammation and Cancer Program, German Cancer Research Center (DKFZ), Heidelberg, Germany; Australian Institute of Tropical Health and Medicine, James Cook University, Cairns, Australia; Infections and Cancer Epidemiology, Infection, Inflammation and Cancer Program, German Cancer Research Center (DKFZ), Heidelberg, Germany; State Key Laboratory of Translational Oncology, Sir YK Pao Centre for Cancer, Hong Kong Cancer Institute, The Chinese University of Hong Kong, Prince of Wales Hospital, Hong Kong SAR, China; Department of Clinical Oncology, State Key Laboratory of Translational Oncology, Sir YK Pao Centre for Cancer, Hong Kong Cancer Institute, The Chinese University of Hong Kong, Prince of Wales Hospital, Hong Kong SAR, China; Department of Radiology, Sun Yat-Sen University Cancer Center, State Key Laboratory of Oncology in South China, Guangzhou, China; Department of Otolaryngology—Head and Neck Surgery, Yong Loo Lin School of Medicine, National University of Singapore, Singapore; State Key Laboratory of Translational Oncology, Sir YK Pao Centre for Cancer, Hong Kong Cancer Institute, The Chinese University of Hong Kong, Prince of Wales Hospital, Hong Kong SAR, China; Department of Clinical Oncology, State Key Laboratory of Translational Oncology, Sir YK Pao Centre for Cancer, Hong Kong Cancer Institute, The Chinese University of Hong Kong, Prince of Wales Hospital, Hong Kong SAR, China; Department of Diagnostic Radiology, Tan Tock Seng Hospital, Singapore, Singapore; Department of Otorhinolaryngology, Head and Neck Surgery, The Chinese University of Hong Kong, Prince of Wales Hospital, Hong Kong SAR, China; Department of Imaging and Interventional Radiology, The Chinese University of Hong Kong, Prince of Wales Hospital, Hong Kong SAR, China; Department of Health Technology and Informatics, The Hong Kong Polytechnic University, Hong Kong SAR, China; Department of Imaging and Interventional Radiology, The Chinese University of Hong Kong, Prince of Wales Hospital, Hong Kong SAR, China; Department of Otorhinolaryngology, Hong Kong Children’s Hospital, Hong Kong SAR, China; The Hong Kong College of Otorhinolaryngologists, Hong Kong SAR, China; Department of Radiation Oncology, Changhua Christian Hospital, Changhua, Taiwan; Genomics Research Center, Academia Sinica, Taipei, Taiwan; Division of Cancer Epidemiology and Genetics, National Cancer Institute, Rockville, MD, USA; Department of Radiation Oncology, Stanford University, Stanford, CA, USA; Clinical Oncology Center, Shenzhen Key Laboratory for Cancer Metastasis and Personalized Therapy, The University of Hong Kong-Shenzhen Hospital, Guangdong, China; Department of Clinical Oncology, The University of Hong Kong, Hong Kong SAR, China; Cancer Research Institute of Zhongshan City, Zhongshan City People’s Hospital, Zhongshan, China; State Key Laboratory of Oncology in South China, Collaborative Innovation Center for Cancer Medicine, Guangdong Key Laboratory of Nasopharyngeal Carcinoma Diagnosis and Therapy, Center for Precision Medicine of Sun Yat-sen University, Sun Yat-sen University Cancer Center, Guangzhou, Guangdong, China; Department of Cancer Prevention, Sun Yat-sen University Cancer Center, Guangzhou, China; State Key Laboratory of Oncology in South China, Collaborative Innovation Center for Cancer Medicine, Guangdong Key Laboratory of Nasopharyngeal Carcinoma Diagnosis and Therapy, Center for Precision Medicine of Sun Yat-sen University, Sun Yat-sen University Cancer Center, Guangzhou, Guangdong, China; Department of Radiation Oncology, Sun Yat-sen University Cancer Center, Guangzhou, Guangdong, China; State Key Laboratory of Translational Oncology, Sir YK Pao Centre for Cancer, Hong Kong Cancer Institute, The Chinese University of Hong Kong, Prince of Wales Hospital, Hong Kong SAR, China; Department of Clinical Oncology, State Key Laboratory of Translational Oncology, Sir YK Pao Centre for Cancer, Hong Kong Cancer Institute, The Chinese University of Hong Kong, Prince of Wales Hospital, Hong Kong SAR, China; Li Ka Shing Institute of Health Sciences, The Chinese University of Hong Kong, Hong Kong SAR, China; Department of Chemical Pathology, The Chinese University of Hong Kong, Prince of Wales Hospital, Hong Kong SAR, China; Centre for Novostics, Hong Kong Science Park, Hong Kong SAR, China; State Key Laboratory of Translational Oncology, Sir YK Pao Centre for Cancer, Hong Kong Cancer Institute, The Chinese University of Hong Kong, Prince of Wales Hospital, Hong Kong SAR, China; Agencia Costarricense de Investigaciones Biológicas (ACIB) - Fundacion del Instituto Costarricense de Investigación y Enseñanza en Nutrición y Salud (INCIENSA), San José, Costa Rica

## Abstract

A meeting of experts was held in November 2021 to review and discuss available data on performance of Epstein-Barr virus (EBV)–based approaches to screen for early stage nasopharyngeal carcinoma (NPC) and methods for the investigation and management of screen-positive individuals. Serum EBV antibody and plasma EBV DNA testing methods were considered. Both approaches were found to have favorable performance characteristics and to be cost-effective in high-risk populations. In addition to endoscopy, use of magnetic resonance imaging (MRI) to investigate screen-positive individuals was found to increase the sensitivity of NPC detection with minimal impact on cost-effectiveness of the screening program.

Based on the evidence presented and discussed at the meeting, a recommendation is made for 1-2 rounds of sex-neutral or male-only screening of middle-aged adults aged 30-69 years in NPC high-risk regions (eg, southern China) using either EBV antibody or plasma EBV DNA testing. A similar recommendation is made for screening of individuals with a family history of NPC in NPC intermediate-risk regions (eg, Southeast Asia). Screening is not recommended in NPC low-risk regions.

Gaps in our understanding of NPC screening, investigation, and treatment methods were identified during the meeting and are summarized in this report. As these gaps are addressed and new data become available, it is expected that the initial recommendations summarized in this report will need to be updated. To this end, we advocate periodic reconvening of the expert panel, with participation by members of head and neck societies and health promotion agencies from NPC high- and intermediate-risk regions.

The incidence of nasopharyngeal carcinoma (NPC) (1) is highly variable across the world. The endemic form of the cancer (nonkeratinizing carcinoma inclusive of both differentiated [World Health Organization type II] and undifferentiated [type III] subtypes) is caused by Epstein-Barr virus (EBV). With growing evidence to support the use of EBV-based biomarkers for NPC screening, there is a need to translate research findings into public health programs for implementation. The goal of EBV-based screening is to detect NPC at an early stage, when available treatments are highly effective and minimize long-term sequelae.

The virtual Nasopharyngeal Cancer Screening Conference (referred to as the meeting henceforth) was held on November 4-6, 2021. Thirty-five experts from various disciplines presented and discussed data on the performance and cost-effectiveness of EBV-based screening for NPC. The objective was to develop recommendations for the use of EBV-based screening tests and for the clinical evaluation and treatment of screen-positive individuals (see also scientific agenda provided in Supplemental Materials, available online). Herein, we summarize our meeting discussions, resultant recommendations, and future research needs.

## Epidemiological factors of NPC to be considered in screening recommendations

### Geographical variation

**Figure 1.**
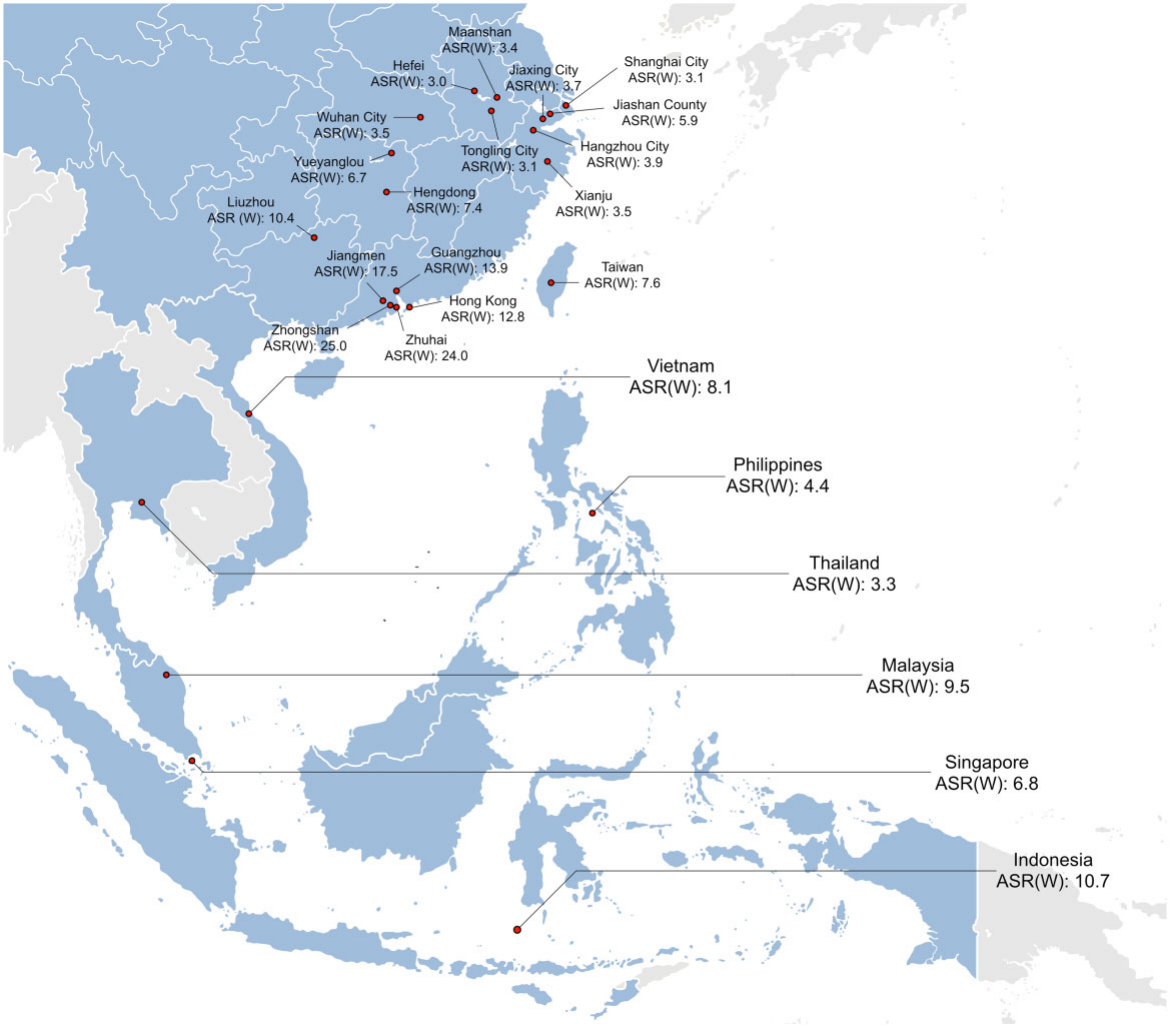
Incidences of nasopharyngeal carcinoma among males from representative high- and intermediate-risk regions in southern parts of China and Southeast Asia. ASR(W) = age-standardized (world) incidence rates.

NPC is characterized by marked geographical variation in incidence (2). Figure 1 summarizes NPC incidences from representative high- and intermediate-risk regions in southern parts of China and Southeast Asia (3-7). The wide variation in NPC incidence is likely to have important implications for the cost effectiveness of NPC screening in different regions of the world.

### Race and ethnicity

In southern China, where the highest rates of NPC have been observed, it has been reported that the Tanka ethnic group historically had a higher risk of NPC than other dialect groups (8). NPC risk in other parts of China and Southeast Asia has been shown to be correlated with the extent of social admixture with southern Chinese (9). The effect of migration has also been studied in high-risk populations migrating to regions of low incidence and vice versa. Results from these studies suggest that genetic and environmental risk factors contribute to the development of this cancer (10). Given the variability in the NPC risk among different ethnic groups, a tailored screening program should be specifically considered in multiethnic countries (eg, Singapore and Malaysia).

### Sex

In endemic regions, a male preponderance of NPC is observed with a male-to-female ratio of 2.5- to 3-fold (2). The lower NPC incidence among females implies a lower cost effectiveness of sex-neutral population screening programs compared with male-only programs.

### Age

In contrast to most other cancers, NPC disproportionately affects middle-aged individuals (11-13). This age-specific incidence pattern needs to be considered when recommendations are made for the target ages for screening.

### Family history

Family history is one of the most recognized risk factors for NPC (12,14-17). Having a first-degree relative with NPC confers a fourfold to tenfold increase in risk of NPC. Targeted screening for this high-risk group (18) might be feasible and cost-effective in some regions where population-based screening is not cost-effective.

### Section summary

NPC has a variable geographic distribution, has a male predominance, preferentially affects middle-aged adults, and is strongly associated with family history. These important characteristics should be considered when making recommendations for screening.

## NPC screening modalities

The pathogenesis of NPC is closely linked to EBV infection (19-21). Therefore, efforts to develop screening for the early detection of NPC have focused on EBV-based tests, including serum anti-EBV antibody and plasma EBV DNA. Two large-scale prospective trials (22-24) (unpublished data; Chen WJ, Yu X, Lu YQ, et al.) (Table 1) that evaluated the use of these EBV-based biomarkers for NPC screening among asymptomatic individuals in Southern China provided much of the data that form the basis for our recommendations. It should be noted that these two trials differ not only in their choice of EBV screening modality but also in their choice of study population, design, and implementation. Direct comparison of results across studies should therefore be avoided.

**Table 1.** Study protocols and study results of the 2 prospective screening trials

Studies	Liu et al. (22), Ji et al. (23), and (Chen et al., unpublished data)^a^	Chan et al. (24)
Test	EBV IgA serology (VCA and EBNA1)	Plasma EBV DNA by PCR
Study design	Cluster randomized controlled trial	Prospective cohort with historical controls
Study population	Male and female, aged 30-59 years (initial phase); male and female, aged 30-69 years (expanded phase)	Male, aged 40-62 years
Number of participants screened	28 680 (initial phase, both sexes); 52 508 (expanded phase, both sexes)^b^	20 174 male participants
Study protocols	EBV IgA testing at recruitment. Participants divided into high-, medium-, and low-risk groups based on a logistic regression model score that combines VCA and EBNA1 testing results. High-risk group defined as screen positive and referred for confirmatory investigations; medium-risk group retested annually, and low-risk group re-screened at 5 years. Screen positivity is defined as high-risk result at baseline testing or high-risk result at retesting.	Two time-point testing protocol: plasma EBV DNA testing at recruitment and retesting offered to participants with positive baseline results at 4 weeks. Participants with both positive baseline and retest results are defined as screen positive and referred for confirmatory investigations.
Sensitivity	1 year of follow-up = 93%; 8 years of follow-up = 75%	1 year of follow-up = 97%
Specificity	1 year of follow-up = 97%; 8 years of follow-up = 95%	1 year of follow-up = 99%
Positive predictive value (PPV)	1 year of follow-up = 4.4%; 8 years of follow-up = 5.1%	1 year of follow-up = 11.0%
Number needed to screen to detect 1 NPC within 1 year of screening.	699	593
Percentage of patients with early stage NPC in the screened group	1 year of follow-up = 68%; 8 years of follow-up = 55%	1 year of follow-up = 70%

a Unpublished data from a manuscript currently under submission by Chen WJ, Yu X, Lu YQ, et al. are included. EBV = Epstein-Barr virus; IgA = immunoglobulin A; NPC = nasopharyngeal carcinoma; PCR = polymerase chain reaction; VCA = viral capsid antigen; EBNA1 = Epstein-Barr nuclear antigen1.

b Of the 175 037 eligible individuals from the towns randomly assigned to screening, 52 508 individuals were screened out.

### EBV antibody-based screening

The differential serological response to EBV between NPC and non-NPC patients forms the basis for the use of EBV antibody biomarkers to screen for NPC (20,21,25). A combined viral capsid antigen and Epstein-Barr nuclear antigen1 (VCA/EBNA1) immunoglobulin A (IgA) antibody score has been developed that uses a logistic regression model (logit *P *=* *-3.934 + 2.203 x IgA anti-VCA + 4.797 x IgA anti-EBNA) to predict NPC and was formally evaluated in a large cluster randomized trial in the Guangdong Province (NCT-00941538). In this trial, 16 towns (initially targeting both men and women aged between 30 and 59 years) were randomly assigned to either EBV antibody screening or to routine care (22). The screening arm consisted of 28 680 individuals recruited of which 3% had high EBV antibody scores (defined as EBV VCA/EBNA1 IgA score ≥ 0.98) and were referred to endoscopic evaluation. In the first year of follow-up, 41 NPC patients were diagnosed of which 38 (93% sensitivity) were detected through EBV VCA/EBNA1 antibody screening. Of the NPC patients, 68% were diagnosed at an early stage (stages I and II). The specificity and positive predictive value (PPV) of the screening test after 1 year of follow-up was 97% and 4.4%, respectively (22).

At the meeting, additional results were presented from an extended trial period in which participation among individuals (age inclusion extended to 30-69 years) from the screening arm was increased to 52 541 individuals, with 8 years of follow-up. After 8 years of follow-up, the sensitivity of EBV antibody testing was 68% (112 screen-detected NPC of 165 confirmed patients). The specificity and PPV were 97% and 6.7%, respectively. Compared with individuals from towns randomly assigned to routine care, NPC mortality among individuals from towns randomly assigned to screening was reduced by 28% overall and by more than 60% for individuals who actually received screening.

An interim analysis conducted at one of the trial sites (Zhongshan) after a median of 4.5 years of follow-up provided information on the risk of NPC among individuals whose initial EBV antibody score was below the 0.98 threshold (23). Of the more than 28 000 individuals with an initial score below 0.98, 16 NPC patients were observed, corresponding to a PPV of less than 1%. This suggests that, among individuals whose initial screening test does not detect high EBV antibody scores, annual rescreening is unlikely to improve the yield of NPC detected.

### Plasma EBV DNA–based screening

The application of plasma EBV DNA testing (26-31) for NPC screening was evaluated in a prospective observational trial, with 20 174 middle-aged men (aged 40-62 years) enrolled, conducted in Hong Kong (24). A 2-timepoint testing protocol (4 weeks apart) of plasma EBV DNA by real-time polymerase chain reaction (rtPCR) was adopted. Participants who had positive results (any detectable levels of plasma EBV DNA) at both initial testing and retesting were defined as screen positive. Participants who had undetectable level of plasma EBV DNA at either the initial or follow-up test were defined as screen negative. Of the screen-positive individuals, 309 (1.5%) were referred for confirmatory investigations including endoscopy and MRI, and 34 NPC patients were identified within 1 year of the initial screen-positive finding. Of these, 70% had early stage disease (stages I or II). The 2-timepoint testing protocol had a sensitivity of 97% for NPC detection and a specificity of 98.5%, with a resultant PPV of 11%. Compared with symptomatic NPC patients from a historical cohort, screening was associated with improved survival (97% 3-year survival vs 72%).

To streamline the testing protocol, a next-generation sequencing (NGS)–based assay that allows for the interrogation of the molecular features of plasma EBV DNA (quantitative and size features) was developed (32). The analysis of molecular features allows the consolidation of 2-timepoint testing into a single timepoint test. On testing individuals from the same screening cohort using plasma collected at baseline, the combined rtPCR and NGS algorithm was shown to improve the specificity and PPV of NPC screening from 98.5% to 99.3% and 11% to 20%, respectively, while maintaining a sensitivity of 97% (32). In an independent study conducted in Taiwan among 798 incident NPC male and female patients and 1746 matched non-NPC participants presented at the meeting, rtPCR testing followed by reflex testing of rtPCR positive samples by NGS led to a predicted sensitivity of 93% (87% for early stage NPC) and specificity of 98% (Lou PJ, in preparation).

Although serum EBV antibody or plasma EBV DNA testing has been shown to be useful for NPC screening, substantial variation has been observed in the performance of these assays across laboratories and using different testing platforms (33,34). Therefore, the results of these large-scale NPC screening studies should not be extrapolated directly to the application of other EBV antibody or DNA assays. Noninferiority and/or bridging studies that formally demonstrate performance of alternative tests should be considered before their use in screening programs. New approaches to complement or replace existing strategies have been proposed (35-38).

### Section summary

Large-scale, prospective trials of adult men and women aged 30-69 years have evaluated EBV antibody and DNA testing for NPC screening. Both approaches had high sensitivity and specificity, led to the detection of NPC at earlier stages, and resulted in improved survival. Evidence for reduced overall NPC mortality was also observed for EBV antibody screening.

## Clinical investigation of screen-positive individuals

### Nasoendoscopic examination

Nasoendoscopic examination is a safe and established investigation for NPC. It allows visualization of the nasopharynx and biopsy of suspicious lesions (39,40). In the 2 large-scale, prospective NPC screening studies described earlier (23,24), nasoendoscopic examination was the core investigation modality for confirming or excluding NPC in test-positiveparticipants.

Screening programs designed to identify small early tumors present challenges for the endoscopists. Narrow band imaging did not improve the diagnostic accuracy as confirmed in a recent meta-analysis study (41,42). Identification of the site(s) for biopsy is problematic for very small tumors. No overall consensus was reached on the endoscopic indications for biopsy in a screening setting, although most would perform a targeted biopsy for mucosal lesions, submucosal bulge, and lymphoid hyperplasia with suspicious asymmetry or focal lesion. There was a consensus that random biopsies of the normal nasopharynx are unlikely to improve lesion detection, especially when magnetic resonance imaging (MRI) is negative.

### Magnetic resonance imaging

MRI is a highly sensitive investigation for NPC detection (43). MRI detects more cancers than endoscopy, especially those early stage cancers hidden from endoscopic view in the pharyngeal recess corner of the nasopharyngeal roof or submucosa (43-47). MRI features also help discriminate NPC from benign hyperplasia and guide the site of biopsy when both entities coexist.

The MRI examination is performed before and after intravenous injection of a standard MRI contrast agent. The MRI examination uses either a short NPC screening protocol with a limited number of sequences targeted to the nasopharynx (46) or a full NPC staging protocol with more sequences and covering the whole head and neck (47). The short screening protocol includes the first groups of nodal spread in the retropharyngeal region and upper internal jugular chain and is converted to a full staging scan if an abnormality is found. Three prospective studies have directly compared the diagnostic performance of MRI and endoscopy (Table 2). The first prospective study was conducted in symptomatic individuals (44,45), and the other 2 studies (46,47) were conducted in the 2 prospective screening cohorts with asymptomatic individuals as described before. MRI demonstrated a superior sensitivity over endoscopy in all 3 studies, detecting nearly all cancers including those cancers that were endoscopy invisible, which ranged from 12% in the symptomatic to 34% in the asymptomatic screening studies (45-47). The high negative predictive value shown by MRI is also valuable in excluding NPC.

**Table 2.** MRI performance for NPC detection; prospective comparative studies with endoscopic examination

			Sensitivity, %		Specificity, %		PPV, %		NPV, %		Accuracy, %	
Publication	Participants	MRI comments	Endo	MRI	Endo	MRI	Endo	MRI	Endo	MRI	Endo	MRI
*AJNR* (45)^a^	Symptomatic	Original MRI	88	100	94	92	91	89	93	100	92	95
	Stage I/II = 37%	4-grade system^b^										
*Ann Oncol* (46)^c^	Asymptomatic	Original MRI	76.5	91.2	97.5	97.5	81.3	83.8	96.7	98.7	94.9	96.7
	EBV DNA screening	4-grade system^d^										
	Stage I/II = 71%											
*Cancer* (47)^e^	Asymptomatic	Original MRI	65.4	100	92.5	86.5	23.0	20.5	98.7	100	91.7	87.0
	EBV-antibody screening	4-grade system^b^	52.6^f^	100^f^								
	Stage I/II = 75%		100^g^	100^g^								
		Updated MRI	65.4	100	92.5	90.2	23.0	25.7	98.7	100	91.7	90.5
		5-grade system^h^										

a Follow-up study of participants in *Radiology* 2011 (44) after minimum 3 years (range 39-86 months, mean = 62 months). EBV = Epstein-Barr virus; Endo = endoscopy; MRI = magnetic resonance imaging; NPC = nasopharyngeal carcinoma; PPV = positive predictive value; NPV = negative predictive value.

b MRI 4-grade system, grading in *Radiology* 2011 (44); grades 3 + 4 = NPC.

c Follow-up study of participants in *N Engl J Med* 2017 (24) after minimum 2 years (24-60 months, median 36 months).

d MRI 4-grade system, grading in *Radiology* 2011 (44); grade 4 = NPC.

e Study included a subgroup that was previously screen negative. Results were from individuals who underwent both examinations (ie, excluded individuals with contraindication to MRI).

f Early stage NPC.

g Late stage NPC.

h Updated MRI 5-grade system in *AJNR Am J Neuroradiol* 2020 (48); grades 4 + 5 = NPC.

Benign hyperplasia can be problematic for MRI, notably causing false-positive findings that reduce MRI specificity. An MRI grading system was therefore introduced (44) and recently updated (48) to detect NPC and differentiate early stage NPC from benign hyperplasia (Table 2). In the latest MRI grading system (48), referral for endoscopic biopsy is indicated in 1) asymmetrical diffuse thickening, which is expansile; 2) loss and/or displacement of the adenoidal stripes; 3) focal lesion, 4) invasion outside the nasopharynx; and 5) metastatic nodes.

Only a small percentage of EBV DNA–screened individuals are positive (ie, high specificity); the difference in the incremental cost-effectiveness ratio (ICER) for screening programs with and without using MRI as a confirmatory test is only about 10%. Adding MRI to the screening program is still considered to be cost-effective (see “Cost-Effectiveness”). However, for NPC screening programs in endemic regions, the lack of MRI resources and radiologists limits the feasibility of performing MRI in all EBV-positive individuals, especially for EBV-antibody screening where a higher rate of false-positive tests leads to more referrals for investigation. Furthermore, where possible, it is desirable to reduce the administration of MRI intravenous contrast agents in normal individuals.

Currently, we suggest contrast-enhanced MRI in individuals with an endoscopic examination that is indeterminate; positive but with a negative biopsy; negative with further EBV positivity after the first round of screening. In the future, the development of a fast, short MRI protocol without a contrast agent could allow MRI to be performed in all EBV screen-positive individuals before endoscopic examination, a positive MRI helping direct the endoscopist to the site of biopsy, and a negative MRI increasing confidence that NPC has not been missed. A short NPC screening protocol without contrast (48) and adjunct value of functional MRI techniques, such as diffusion-weighted imaging to discriminate early stage NPC and benign hyperplasia, were presented and discussed (49-51). Artificial intelligence using deep convolutional neural networks (52,53) offers a glimpse into the future with the potential to automatically assess fast plain MRI scans and generate reports for screening programs.

### Section summary

Confirmation of NPC in screen-positive individuals is by nasopharyngeal endoscopic examination and biopsy. Given the higher sensitivity of MRI in detecting early stage NPCs, MRI should be considered in addition to endoscopic examination.

## Follow-up of individuals without an immediate diagnosis of NPC

The formulation of a clinical follow-up plan should consider the follow-up modalities, frequency of follow-up, and when to terminate the follow-up. However, there is a paucity of evidence to support best practice, so the suggestions below are based on the limited data, experience, and discussions during the meeting.

A high risk of NPC among screen-positive individuals has been shown in the initial years following a screen-positive result. In one key study that followed a group of 1445 asymptomatic individuals with a moderate or high serum EBV antibody score (defined as EBV VCA/EBNA1 IgA score ≥ 0.65) in the Guangdong NPC screening trial (54), a total of 38 NPC cases were identified over the course of 4 years. Of these 38 NPC cases, the majority (ie, n = 25, 66%) identified at the time of the initial positive screen, 12 NPC cases in the first year follow-up, and 1 NPC case in the second year follow-up. No NPC cases were identified 4 years after the initial screen. The findings therefore support more frequent follow-up in the initial 1-2 years after a screen-positive result.

## For screen-positive, asymptomatic individuals with negative nasoendoscopy and MRI

### What to do during follow-up

Endoscopy is useful for detecting an emerging small NPC during follow-up. Interval nasopharyngeal endoscopy with photo documentation would facilitate recognition of subtle changes. EBV-based biomarkers could be repeated to see if the status (positive vs negative) has changed. Specifically, there is a lack of data on the complementary roles of EBV-based biomarkers, for example, whether plasma EBV DNA could help differentiate those who need follow-up among EBV antibody screen-positive subjects. For individuals with persistent positive EBV-based testing results, MRI is useful for excluding small, concealed tumors. For those who have already undergone MRI or for those with a suspicious abnormality on the initial MRI without biopsy-proven NPC, follow-up MRI using a short screening protocol without contrast can evaluate interval changes indicative of disease.

### Optimal timing and frequency of follow-up

The optimal timing and frequency for follow-up is unknown, but in general, more frequent follow-up (eg, 6 monthly) should be arranged in the first year when most NPCs are diagnosed.

### Termination of follow-up

As available data suggest an elevated risk of NPC for the initial 1-2 years following a positive screen, the termination of follow-up could be considered 2 years after the screening test. Follow-up may need to be extended to 3-4 years if MRI is not available (45,46). Given the high negative predictive value (NPV) of MRI, future studies may determine if EBV tests alone are sufficient for follow-up when the initial endoscopy and MRI are negative.

## For screen-positive, asymptomatic individuals with a suspicious abnormality on nasoendoscopy or MRI but no diagnosis of NPC

For individuals without biopsy-proven NPC but indeterminate findings or suspected small NPCs on endoscopy or MRI, consensus was that a tailored clinical follow-up plan should be offered. Of note, 2 comparative follow-up studies after a minimum of 2-3 years found 4 more NPCs in individuals with an initial negative endoscopic examination and positive MRI. These were small NPCs mostly located deep in the pharyngeal recess, which grew slowly on MRI surveillance and became endoscopically evident after 36-43 months (45,46).

### Section summary

Participants with positive EBV-based blood test results but 1) negative endoscopy and/or MRI or 2) an abnormality on endoscopy and/or MRI without biopsy-proven NPC should be followed up. EBV blood tests, endoscopy, and MRI are all modalities for follow-up. Future studies are needed to evaluate the potential complementary roles of EBV antibody and plasma EBV DNA in this setting. Frequency of follow-up should be greater in the first year, and termination of follow-up can be considered after 2 years if all tests are negative.

## Cost-effectiveness of NPC screening in endemic areas

Determining the cost effectiveness of any cancer screening program is important before its implementation. Using the ICER threshold of U$50 000/quality-adjusted life year [to define highly cost-effective interventions in high-income countries (55)] or a willingness to pay (WTP) threshold of double a country’s annual gross domestic product (GDP) per capita (56), once-lifetime sex neutral screening of middle-aged adults is generally found to be cost-effective in high-risk endemic regions of the world (57). In countries with intermediate NPC incidence, screening of high-risk subgroups may sometimes be cost-effective. Ultimately, WTP thresholds are arbitrary, and ICERs must be considered in context of other local public health interventions.

Supplementary Table 1 (available online) summarizes the modeled cost-effectiveness of screening males and females aged 50 years in representative locations within Southeast Asia using different screening approaches and within selected risk subgroups. As shown in the Supplementary Table 1 (available online), once-lifetime, sex-neutral screening meets the ICER and WTP thresholds defined above in nearly all scenarios presented. Serum EBV antibody and plasma EBV rtPCR approaches were shown to be cost-effective. The inclusion of MRI to complement endoscopy among screen-positive individuals only marginally affects cost-effectiveness given the small fraction of screened individuals requiring such intervention and the trade-off between MRI costs and higher sensitivity of NPC detection. Male-only screening is more cost-effective than female or sex-neutral screening, as expected given the higher burden of NPC among males. Cost effectiveness is also likely to decrease with an increasing number of lifetime screens.

Once-lifetime sex-neutral NPC screening of middle-aged adults using EBV-based biomarkers in endemic areas is likely to be cost-effective using common WTP thresholds. Incorporation of MRI procedures to complement endoscopy for screen-positive individuals only marginally impacts cost-effectiveness. Cost-effectiveness can be enhanced by screening individuals with increased risk of NPC, particularly in regions with intermediate NPC incidence.

## Summary recommendations for NPC screening and management of screen-positive individuals

**Figure 2.**
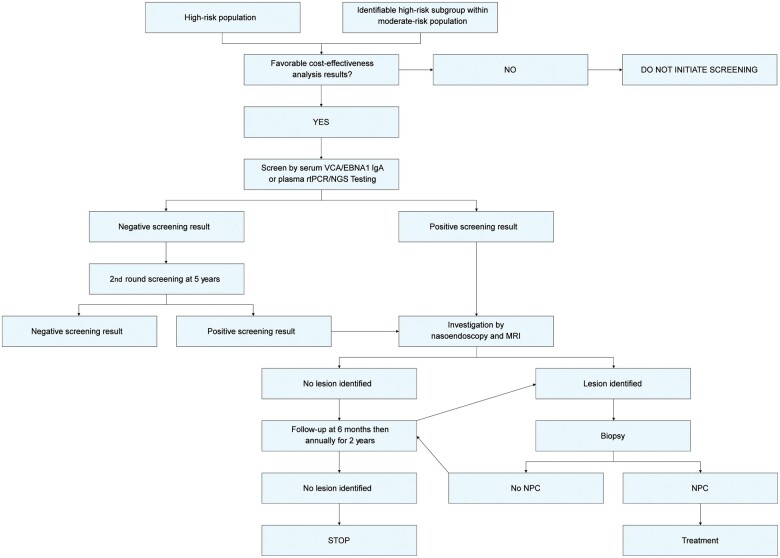
Summary of screening recommendations. IgA = immunoglobulin A; MRI = magnetic resonance imaging; NGS = next-generation sequencing; NPC = nasopharyngeal carcinoma; rtPCR = real-time polymerase chain reaction; VCA/EBNA1 = viral capsid antigen and Epstein-Barr nuclear antigen1.

Based on the above, the following recommendations are made (Figure 2).

### Target population for NPC screening

- Sex-neutral or male-only screening for middle-aged adults (ages 30-69 years) in high-risk endemic areas;
- Screening for middle-aged adults with a family history of NPC in intermediate-risk areas;
- One round of screening followed by another round 5 years later has been shown to be cost-effective and could be considered.

### Screening modalities of choice

- Combined EBV antibody testing for anti-EBV VCA/EBNA1 IgA [using assays harmonized with those used in the Liu et al. and Chen et al. studies (22,38) and its associated score at a referral threshold of 0.98];
- Plasma EBV DNA by PCR [using an assay harmonized with the one used in the Chan et al. and Le et al. studies (24,34)];
- Reflex testing of rtPCR-positive samples by NGS could be considered to improve specificity;
- Alternative assays should only be used after they have been formally bridged and harmonized to the original assays used in the formal trials.

### Investigation of screen-positive individuals

Investigation should ideally include endoscopic and MRI evaluation:

- Endoscopy with a targeted biopsy approach without any sampling biopsies
- MRI followed by referral for endoscopic biopsy if NPC is suspected

### Follow-up of screen-positive individuals without an immediate diagnosis of NPC

- Follow screen-positive, investigation-negative individuals at 6-month intervals in the first year and then reassess once in the second year by rescreening via endoscopy, repeat EBV-based tests, and MRI (if the EBV-based test is persistently positive and MRI has not been performed initially).
- Follow screen-positive, investigation-positive (endoscopic or MRI) individuals dependent on investigation findings.

### Treatment of screen-detected NPC patients

- Treatment as per current practice for nonscreen-detected patients based on TNM staging

## Knowledge gaps and future research priorities

Here, we summarize the key knowledge gaps identified during the meeting to help define priorities for research in this area in the coming years. Although we avoid prescribing specific studies to address existent gaps, there is a consensus that, to the extent possible, randomized clinical trials should be considered to address these important knowledge gaps.

With sufficient evidence to support the implementation of screening programs, subsequent monitoring of short-term (incidence, stage distribution) and long-term (morbidity, overall mortality) impact of the programs will be important. We therefore recommend that plans for monitoring the impact of NPC screening programs be developed and implemented in parallel with the development and implementation of the screening programs themselves.

### Unanswered questions on screening for the early detection of and morbidity and/or mortality reduction from NPC

Serum antibody and plasma DNA EBV have been shown to be highly sensitive tests to screen for prevalent, asymptomatic early stage NPC. However, no formal head-to-head comparison of these tests has been performed in a large study cohort except one for evaluation among high-risk individuals with a positive family history of NPC (58). Future studies should consider concurrent testing to allow for direct comparisons between alternative screening strategies, including their cost-effectiveness. These studies might also address the potential complementary roles of the 2 EBV-based biomarkers.

Initial recommendations made above focus on 1-time or at most 2-time screening efforts. The interval at which screening should be repeated is not well understood and requires further formal evaluation.

### Unanswered questions on best practices for the diagnosis of NPC and follow-up among screen-positive individuals

As alluded to above, one key component to the ultimate success of an NPC screening program is the ability to detect early stage NPC in asymptomatic individuals for whom treatment has been shown to be highly successful and to limit morbidity. MRI has clearly demonstrated its superior sensitivity for early NPC detection, however, obstacles remain to the use of MRI procedures for this purpose. Accessibility to MRI resources is limited in some settings. To this end, simplified, rapid MRI protocols for plain scans without contrast tailored for the screening purpose have been proposed. Evaluation of the performance of such a simplified MRI protocol should be the focus of future research in this area. Such testing arrangement could allow screen-positive subjects to undergo MRI first before endoscopy, so results can be used to prioritize referral in a screening setting and help guide biopsy of suspicious sites.

Regarding the management of screen-positive but endoscopy- and MRI-negative individuals, the roles of EBV-based testing, endoscopies, and MRI as follow-up modalities have to be delineated in future research. The goal is to minimize unnecessary procedures and potentially expensive and time-consuming follow-up.

### Mechanism to monitor and incorporate new research findings into revised NPC screening and management recommendations

The recommendations for NPC screening and management put forth herein are based on current knowledge and data available. A mechanism to ensure periodic review of new research findings and updating of NPC screening and management recommendations is needed. Also, it is important to stress that new cost-effectiveness analyses will be required. We intend to reconvene the expert panel of this meeting every 3-5 years to review new data in the field.

## Supplementary Material

djad012_Supplementary_Data

## Data Availability

The data underlying this article (ie, recordings of the meeting and discussion) will be shared on reasonable request to the corresponding author.

## Notes

**Role of the funder:** The sponsors did not play a role in the content of this commentary, the writing of the manuscript, or the decision to submit this manuscript for publication.

**Disclosures:** WKJL holds equities in Grail/Illumina. BBYM serves in the advisory board and receives speaker’s honorarium from Novartis, Bristol-Myers Squibb and MSD and receives research grant from Novartis and is a consultant to Viracta Therapeutics and Y-Biologics. EPH receives speaker’s honoraria from Merck Sharp & Dohme (MSD) and Merck Serono and serves as a consultant or in the advisory board from MSD. QTL is a consultant to MSD and serves in the advisory boards of Nanobiotics, Roche and Coherus. ATCC receives research funding from Merck Serono, MSD, Novartis, Pfizer and Eli Lily, speaker’s honorarium from Beigene, Springer and Pfizer, travel funding from BMS, MSD, Pfizer, Roche, Novartis and AstraZeneca, and is an advisory board member for MSD, Tessa Therapeutics. KCAC holds equities in DRA, Take2, and Grail/Illumina and was a consultant to Grail. KCAC previously received research funding from Grail.

**Author contributions:** WKJL: Conceptualization, Writing—original draft; Writing—review and editing; ADK: Conceptualization, Writing—original draft, Writing—review and editing; JAM, ZL, KJY, MLKC, BBYM, MYC, BAP, PJL, JKSW, WLH, JS, DLD, TW, EPH, HL, RKT, KCWW, JPG, ACV, QYA, LMW, VA, JCL, CJC, RMP, QTL, AWML, MJ, SC, JM, ATCC: Writing—review and editing; KCAC: Conceptualization, Writing—original draft; Writing—review and editing; AH: Conceptualization, Writing—original draft; Writing—review and editing.
