# Supplementary material for: Recommendations for Epstein-Barr virus–based screening for nasopharyngeal cancer in high- and intermediate-risk regions
Source: J Natl Cancer Inst. 2023 Feb 1;115(4):355–64. doi: 10.1093/jnci/djad012 (PMC10086631; doi:10.1093/jnci/djad012)
Supplement: djad012_Supplementary_Data [file djad012_supplementary_data.docx]

**Supplementary Material**

**Supplementary Table 1. Summary of incremental cost effectiveness ratios (ICERs) across selected Asian populations, sex, population subgroups and screening strategies.** The ICER threshold of U$50,000/QALY is considered as cost-effective. Once-lifetime, sex-neutral screening meets the ICER and willingness-to-pay (WTP) thresholds in nearly all scenarios among 50-year old males and females in representative locations within Southeast Asia.

Abbreviations: ICERs, incremental cost effectiveness ratios; PPP, purchasing power parities; QALY, quality adjusted life year; PCR, polymerase chain reaction (i.e. EBV DNA analysis by PCR); Ab, antibody (i.e. EBV serology), Endo, endoscopy; NP PCR, nasopharyngeal swab EBV DNA polymerase chain reaction testing; FH, family history.

Ab/Endo means the use of EBV antibody testing followed by endoscopy to confirm NPC among screen-positive cases. PCR/Endo means the use of rtPCR-based plasma EBV DNA testing followed by endoscopy to confirm NPC among screen-positive cases. PCR/Endo/MRI means the use of rtPCR-based plasma EBV DNA testing followed by endoscopy and MRI to confirm NPC among screen-positive cases. Ab/NP PCR/ Endo means the use of EBV antibody testing followed by EBV DNA testing in nasopharyngeal swab and then endoscopic confirmation of NPC status.
